# Analysis of type I IFN response and T cell activation in severe COVID-19/HIV-1 coinfection: A case report

**DOI:** 10.1097/MD.0000000000021803

**Published:** 2020-09-04

**Authors:** Gabriella d’Ettorre, Gregorio Recchia, Marco Ridolfi, Guido Siccardi, Claudia Pinacchio, Giuseppe Pietro Innocenti, Letizia Santinelli, Federica Frasca, Camilla Bitossi, Giancarlo Ceccarelli, Cristian Borrazzo, Guido Antonelli, Carolina Scagnolari, Claudio Maria Mastroianni

**Affiliations:** a Department of Public Health and Infectious Diseases, Sapienza University of Rome, Viale del Policlinico 155; b Laboratory of Virology, Department of Molecular Medicine, affiliated to Istituto Pasteur Italia - Cenci Bolognetti Foundation, Sapienza University; c Microbiology and Virology Unit, Hospital “Policlinico Umberto I”, Sapienza University, Rome, Italy.

**Keywords:** human immunodeficiency virus-1, interferon, protease inhibitor, severe acute respiratory syndrome coronavirus-2, T cell activation

## Abstract

**Rationale:**

Complex immune dysregulation in interferon (IFN) and T cell response has been observed in human immunodeficiency virus (HIV-1)-infected patients as well as in coronavirus disease-2019 (COVID-19) patients. However, severe acute respiratory syndrome coronavirus-2 (SARS-CoV-2)/HIV-1 coinfection has been described in only few cases worldwide and no data are available on immunological outcomes in HIV-1-patients infected with SARS-CoV-2. Hence, this study aims to compare type I IFN response and T cell activation levels between a SARS-CoV-2/HIV-1-coinfected female patient and age-matched HIV-1-positive or uninfected women.

**Patient concerns:**

A 52-year-old woman diagnosed with SARS-CoV-2/HIV-1 coinfection, ten HIV-1-positive women and five age-matched-healthy individuals were enrolled in this study.

**Diagnoses:**

SARS-CoV-2 infection caused severe pneumonia in the second week of illness in HIV-1-positive patient under protease inhibitors. Chest high-resolution computed tomography images of the SARS-CoV-2/HIV-1-coinfected patient showed bilateral ground-glass opacities.

**Interventions:**

SARS-CoV-2/HIV-1-coinfected female patient under darunavir/cobicistat regimen received a 7-days hydroxychloroquine therapy. Analysis of IFN*α*/β mRNA levels and CD4 and CD8 T cell (CD38, human leukocyte antigen-DR [HLA-DR], CD38 HLA-DR) frequencies were performed by RT/real-time PCR assays and flow cytometry, respectively. Median relative difference (MRD) was calculated for each immunological variable. For values greater than reference, MRD should be a positive number and for values that are smaller, MRD should be negative.

**Outcomes:**

The severe pneumonia observed in SARS-CoV-2/HIV-1-positive patient under protease inhibitors was reversed by a 7-days hydroxychloroquine therapy. At the end of treatment, on day 7, patient reported resolution of fever, normalization of respiratory rate (14 breaths/min), and improved oxygen arterial pressure with a F_i_O_2_ of 30%. MRD values for IFN*α*/β and CD4 and CD8 T cells expressing CD38 and/or HLA-DR found in SARS-CoV-2-/HIV-1-coinfected woman were approximatively equal to 0 when refereed respectively to HIV-1-positive female patients [MRDs IFN*α/*β: median −0.2545 (range: −0.5/0.1); T cells: median −0.11 (range: −0.8/1.3)] and ≥ 6 when referred to healthy individuals [MRDs IFN*α/*β: median 28.45 (range: 15/41.9); T cells: median 10 (range 6/22)].

**Lessons:**

These results indicate that SARS-CoV-2 infection in HIV-1-positive female patient was associated with increased levels of IFNα/β-mRNAs and T cell activation compared to healthy individuals.

## 1 Introduction

The novel coronavirus disease 2019 (COVID-19), a contagious acute respiratory syndrome, has spread worldwide from China, causing more than 200,000 deaths, as of May 2020.^[1]^ Despite high number of people living with human immunodeficiency virus (HIV)-1 globally (about 37 million) and higher severity impact for certain viral infections in this category,^[2]^ severe acute respiratory syndrome coronavirus-2 (SARS-CoV-2)/HIV-1 coinfection has been described in few cases.^[3–6]^ Furthermore, black people are overrepresented among reported COVID-19 cases and deaths, especially in some areas of US.^[7]^

Chronic immune activation and persistent interferon (IFN)-I response are well-known driver of HIV-1 disease progression.^[8,9]^ Notably, SARS-CoV-2 mono-infection has been also associated to alteration in IFN*α*/β and T cell immune activation,^[10,11]^ suggesting that HIV-1 and SARS-CoV-2 might both promote deleterious immunological and clinical consequences.

This study reports a severe case of SARS-CoV-2 in a black female patient co-infected by HIV-1 under protease inhibitors (PI) regimen, who was treated with hydroxychloroquine. To address the role of IFN-I response and T cell activation in SARS-CoV-2/HIV-1 coinfection, we have analyzed IFN-I transcript levels and frequencies of CD4 and CD8 T cells expressing CD38 and/or human leukocyte antigen-DR [HLA-DR] in the SARS-CoV-2/HIV-1-coinfected subject, compared to those of HIV-1-monoinfected patients and healthy blood donors.

## 2 Methods

### 2.1 Participants

**Figure 1:**
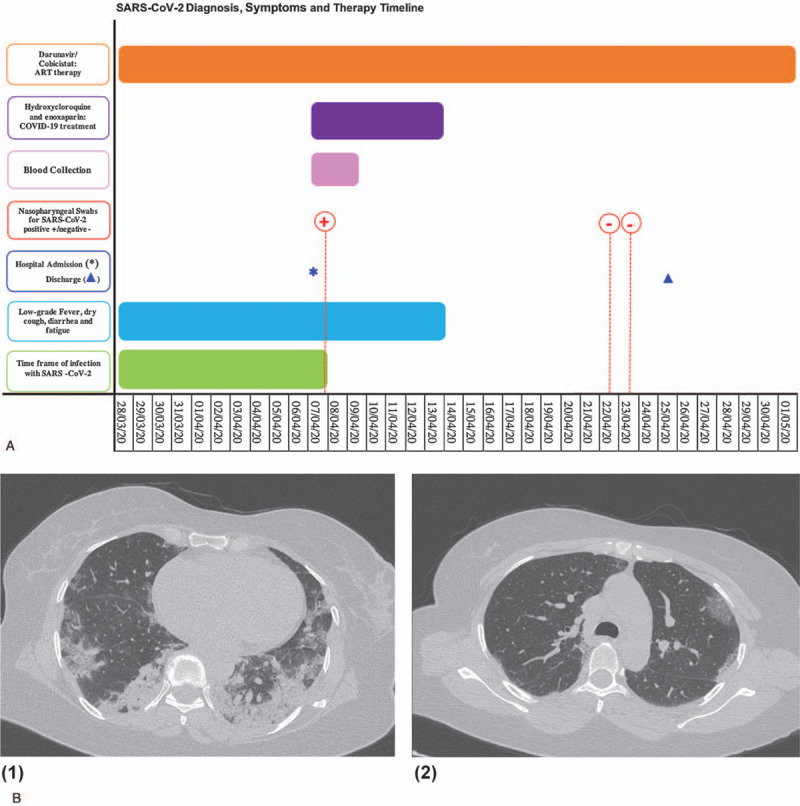
Panel A. Diagnosis, symptoms, and therapy timeline in the SARS-CoV-2/HIV-1-coinfected patient. Red dotted line: nasopharyngeal swabs. Blue asterisk: Hospital admission; Blue triangle: Hospital discharge. Panel B1-B2. Representative chest High-resolution computed tomography (HRCT) images of the SARS-CoV-2/HIV-1-coinfected patient, detected bilateral ground-glass opacities.

A black Ethiopian 52-year-old woman with SARS-CoV-2/HIV-1 coinfection was admitted to Department of Infectious Diseases at Policlinico Umberto I, Hospital of Sapienza University of Rome (Italy) during the epidemic wave in Italy. Nasopharyngeal swabs were collected within 48 hours of hospital admission and before hospital discharge (Fig. 1, Panel A). Blood samples were also collected from SARS-CoV-2-positive patient (Fig. 1, Panel A), age-matched virologically suppressed HIV-1-infected women (n = 10) and healthy individuals (n = 5). The study was approved by the institutional review board (Ethics Committee of Umberto I General Hospital, Rome, approval number/ID Prot. 109/20209). All study participants gave written informed consent.

### 2.2 RT-qPCR detection of SARS-CoV-2 RNA

Viral RNA was extracted from nasopharyngeal swabs using Versant SP 1.0 Kit (Siemens Healthcare Diagnostics). Ten μl of extracted RNA was reverse-transcribed and simultaneously amplified using a Real time RT PCR system (RealStar SARS-CoV2 RT PCR, Altona Diagnostics), targeting E and S viral genes.

### 2.3 Flow cytometry assay

Phenotypes and activation markers were evaluated by Miltenyi Biotec flow cytometer-MACSQuant Analyzer on lysed and washed whole-blood samples by the following antihuman monoclonal antibodies: CD3-PerCP, CD4-APC-Vio770, CD8-FITC, CD38-APC and HLA-DR-PE (Miltenyi Biotec, Bergisch Gladbach, Germany). Gating analysis and data were analyzed using MACSQuantify software 2.5 (Miltenyi Biotec).^[12]^

### 2.4 Real-time polymerase chain reaction assay

Total RNA was extracted using Total RNA Purification kit (Norgen, Thorold, Canada) and reverse transcribed using High Capacity cDNA Reverse Transcription Kit (Applied Biosystems, USA). Relative quantification of IFN*α*, IFNβ and housekeeping gene β-glucuronidase target mRNA expression levels were carried out with LightCycler 480 instrument (Roche, Basel, Switzerland), as previously described.^[13]^

### 2.5 Statistical analysis

Data on IFNs gene expression level and activated T cells frequencies were expressed as median relative difference (MRD), as previously reported.^[14]^ In particular, MRD was defined as *MRD* = Δ/*X*_2_=(*X*_1_–*X*_2_)/*X*_2_ where *X*_1_ is the level of immunological measurements, expressed as median, in the SARS-CoV-2/HIV-1-coinfected patient, while *X*_2_ is measure of the same variables referred to age-matched HIV-1-positive female patients or healthy women. For values greater than reference, MRD should be a positive number (Δ > 0) and for values that are smaller, MRD should be negative (Δ < 0). MRD with 1 sigma is uncertain on the meaning for each parameter.

## 3 Case report

### 3.1 Patient

A 52-year-old woman with SARS-CoV-2/HIV-1 coinfection under darunavir/cobicistat regimen was analyzed. SARS-CoV-2 diagnosis, symptoms and therapy timeline is shown in Figure 1, Panel A. For the past 23 years, her HIV-1 status has been immunologically and virologically stable with no reported opportunistic diseases. Laboratory test results at admission time revealed a HIV-1 viral load below level of detection (<37 HIV-1 RNA copies/ml) in line with results of past months and a CD4 T cell count of 242 cells/μl, CD8 T cell count of 336 cells/μl, with a CD4/CD8 T cells ratio of 0.72. Besides her HIV-1 infection diagnosed in 1997, she had a 15 pack-year smoking history, sobriety from alcohol and no intravenous drug abuse habits. At hospital admission, the patient was febrile (37.5°C), with a blood pressure of 120/70 mm Hg, a pulse of 120 beats/min, a peripheral blood saturation of 96%, a respiratory rate of 24 breaths/min and Glasgow Coma Scale of 15. Furthermore, the patient had a normal total leucocyte count (7210 cells/μl) with an absolute lymphocytopenia (610 cells/μl), elevated PCR (15.67 mg/dl), interleukin-6 (50.96 pg/ml) and normal PCT value (0.09 ng/ml). Arterial blood gas analysis showed a pH of 7.48, a value of 35 mm Hg for CO_2_ arterial pressure and 70 mm Hg for O_2_ arterial pressure, a P/F ratio of 333 mm Hg, based on a F_i_O_2_ of 21%. *M. pneumoniae* IgM and *C. pneumoniae* IgM on blood test resulted negative, as peripheral blood smear, TB-GOLD QuantiFERON and urinary antigen of *Legionella*. Thus, she took a nasopharyngeal swab, resulted positive for PCR amplification of SARS-CoV-2 E and S viral genes. She, then, performed a thorax high-resolution computed tomography scan that detected multiple peripheral ground-glass areas bilaterally and rare crazy paving signs, highly suspicious of COVID-19 pneumonia (Fig. 1, Panel B_1_-B_2_).

Hence, she underwent high flow nasal cannula with a F_i_O_2_ of 50% and 60 l/min, associated with a 7-days therapy based on hydroxychloroquine 200 mg bid and enoxaparin 4000 UI bid, maintaining darunavir/cobicistat. At the end of treatment, on day 7, patient reported resolution of fever, amelioration of her fatigue and normalization of respiratory rate (14 breaths/min), requiring less intensive oxygen support. Then, she interrupted hydroxychloroquine. Contemporarily, she took an arterial blood gas analysis that showed improved oxygen arterial pressure (98 mm Hg) with a F_i_O_2_ of 30%. Besides, her PCR declined (2.1 mg/dl) and lymphocytes increased (800 cells/μl). On day 14, patient interrupted oxygen support with normalization of arterial blood gases pressures in ambient air and performed 2 consecutive nasopharyngeal swabs 24 hours apart resulted negative. Lymphocyte count was repeated with detection of lymphocytopenia (810 cells/μl), CD4 T cell count 528 cells/μl, CD8 T cell count 533 cells/μl and a CD4/CD8 T cell ratio of 0.99.

### 3.2 Type I IFN and T cell activation

**Figure 2:**
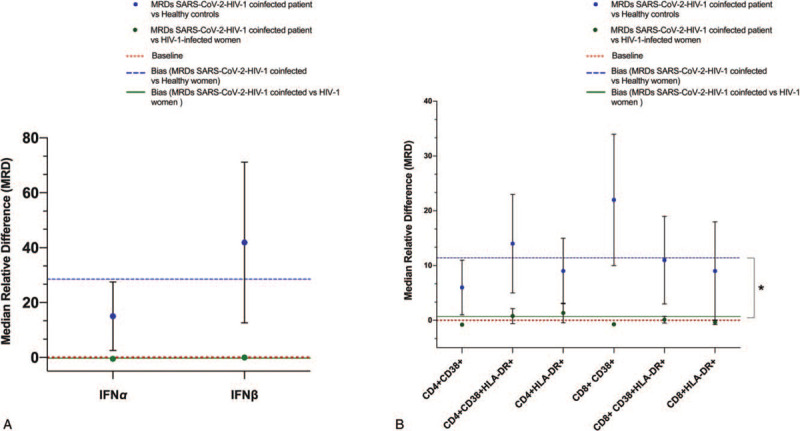
MRDs in IFNα and IFNβ gene expression (Panel A) and T cell immune activation (Panel B) levels between the SARS-CoV-2/HIV-1-coinfected patient and HIV-1-positive female patients (green-filled circle) or healthy women (blue-filled circle). SARS-CoV-2/HIV-1-coinfected patient data are representative of 3 independent experimental replicates and results are provided as median for the entire specimen. Vertical bars indicate the errors. The errors on the MRD are calculated with standard error propagation. The red dotted line (Baseline) markers the case of zero difference between variables measurement in the SARS-CoV-2/HIV-1-coinfected patient and HIV-1-positive female patients or healthy women. The blue dotted line represents bias for MRDs between variables measurement in the SARS-CoV-2/HIV-1-coinfected patient and healthy women, whereas the green continuous line indicates bias for MRDs between variables measurement in the SARS-CoV-2/HIV-1-coinfected patient and HIV-1-positive female patients. HIV-1 = human immunodeficiency virus-1, IFN = interferon, MRD = median relative differences, SARS-CoV-2 = severe acute respiratory syndrome coronavirus-2.

Because of the key role of chronic immune activation and persistent IFN-I response in driving HIV-1 disease,^[8,9]^ we evaluated IFN*α* and IFNβ gene expression and T cell activation levels in patient with SARS-CoV-2/HIV-1 coinfection. MRDs of IFN*α* and IFNβ mRNAs and T cell activation levels were calculated referring to age-matched HIV-1-positive female patients and healthy women. We found that MRDs for IFN*α* and IFNβ and T cells were on average approximatively equal to 0 when refereed to HIV-1-positive female patients [MRDs IFN*α/*β: median −0.2545 (range: −0.5/0.1); T cells: median −0.11(range: −0.8/1.3) (Fig. 2, Panel A, B)]. By contrast, MRDs values of IFNs and T cells immune activation were always ≥6 when referred to healthy individuals [MRDs IFN*α/*β: median 28.45 (range: 15/41.9); T cells: median 10 (range 6/22) (Fig. 2, Panel A, B)].

## 4 Discussion

Little is known about clinical and immunological outcomes of HIV-1-patients infected with SARS-CoV-2.^[15]^ Our case report is consistent with previous researches that described progression of SARS-CoV-2 viral pneumonia in the second week of illness.^[16]^ Furthermore, as reported in a study by Thevarajan et al,^[17]^ peripheral CD4 and CD8 T cell count increases during host immune response, despite CD4/CD8 T cell ratio was dissimilar to the one found in the same case reported. At the present, COVID-19 in people living with HIV-1 has been described in few cases in China,^[3,4]^ in a case series of 5 patients from Spain,^[5]^ with just 2 patients on PI based regimen and in a retrospective Italian study, with an 11% of patients receiving PI.^[15]^

PI drugs, especially lopinavir/ritonavir have been considered, together with hydroxychloroquine, as first-line drug in COVID-19 therapeutic regimens. Darunavir/cobicistat and darunavir/ritonavir have also been included as alternative regimen to lopinavir/ritonavir by the Italian society in infectious and tropical diseases (SIMIT), especially in case of intolerable side effects.^[18]^

However, a recent clinical trial showed that lopinavir/ritonavir was ineffective as a monotherapy against SARS-CoV-2 pneumonia.^[19]^

Since in vitro activity against SARS-CoV and Middle East respiratory syndrome CoV (MERS-CoV) was reported for PI drugs in combination with IFNβ,^[20,21]^ it is reasonable to suppose existence of a synergistic PI and IFNβ effect against SARS-CoV-2, as also suggested by stronger efficacy of combined IFNβ, lopinavir-ritonavir and ribavirin treatment compared to lopinavir/ritonavir alone in patients with COVID-19.^[22]^

Thus, the aim of this report is suggesting further investigation on efficacy of treatment in patients with COVID-19, especially in potential prevention and earlier stages of disease.

In addition, we presented a severe case of COVID-19 in a black person who recovered without serious complications. It is reported in literature how non-Hispanic black people, especially in the US, seem to have higher mortality rate, compared to white persons,^[23]^ even though more studies would be needed in this field. Blacks are disproportionately affected by poverty, mass incarceration, infant mortality, limited health care access, and health-related conditions including heart disease, diabetes, stroke, kidney disease, respiratory illness, and HIV-1. All these facts and the tendency of black African people to live in close and numerous communities seem to increase the exposure risk of this population.^[24]^

Interestingly, our results also indicate, for the first time to our knowledge, that SARS-CoV-2/HIV-1-coinfected woman had levels of IFN*α*/β and activated CD4 and CD8 T cells higher than those recorded in healthy donors, but comparable to those found in HIV-1-monoinfected women. Levels of HLA-DR CD38 CD4 and HLA-DR CD38 CD8 T cells have been reported to be increased in SARS-CoV-2-infected patients^[17]^ together with common inflammation cytokines.^[25]^ Moreover, it has found that SARS-CoV-2 triggers lower levels of IFN-I/III in ex vivo human lung tissue explants from cancer patients.^[26]^ It has also showed that low IFN-I-III levels are produced during SARS-CoV-2 infection despite a consistent chemokine signature in cell lines, primary cell cultures, ferrets and COVID-19 patients.^[27,28]^ Then, our findings suggest that SARS-CoV-2 infection did not adversely affect IFN*α*/β and activated T cells levels in HIV-1-infected patients.

In summary, we found that SARS-CoV-2 infection can cause severe disease in HIV-1-positive patient under PI regimen. The outcome of COVID-19 was reversed by a 7-days hydroxychloroquine therapy, although, to date, there have been no robust clinical trials that have shown efficacy of these agents for this illness.^[29]^ Notably, active SARS-CoV-2 infection was not associated with increased levels of IFN*α* and IFNβ gene expression and T cell immune activation compared to HIV-1-monoinfected women. Although SARS-CoV-2/HIV-1-coinfected patient had no significant alterations in levels of immune dysregulation compared to HIV-1-positive patients, caution must be exercised because analysis was performed in only one HIV-1-coinfected patient. Studies with larger patient series, currently under way, will be needed to explore this topic.

Consent for publication: Informed written consent was obtained from the patient for publication of this case report.

## Author contributions

CP, GPI, and CB wrote the paper, carried out the experiment and performed statistical analysis. LS, FF, and CB collected the samples and participated in carrying out the experiments. GR, MR, GS, GD, and GC provided patient's samples and participated in the design and revision of the manuscript. GA and CMM participated in the design and revision of the manuscript. GD and CS conceived the study, analyzed the data, wrote the paper and supervised the work. All authors reviewed the work and approved the final manuscript.
