# Analysis of type I IFN response and T cell activation in severe COVID-19/HIV-1 coinfection: A case report: Erratum

**DOI:** 10.1097/MD.0000000000022949

**Published:** 2020-10-16

In the article, “Analysis of type I IFN response and T cell activation in severe COVID-19/HIV-1 coinfection: A case report”,^[1]^ which appears in Volume 99, Issue 36 of *Medicine*, panel a of Figure 1 appeared incorrectly and has been updated to the below:

**Figure 1:**
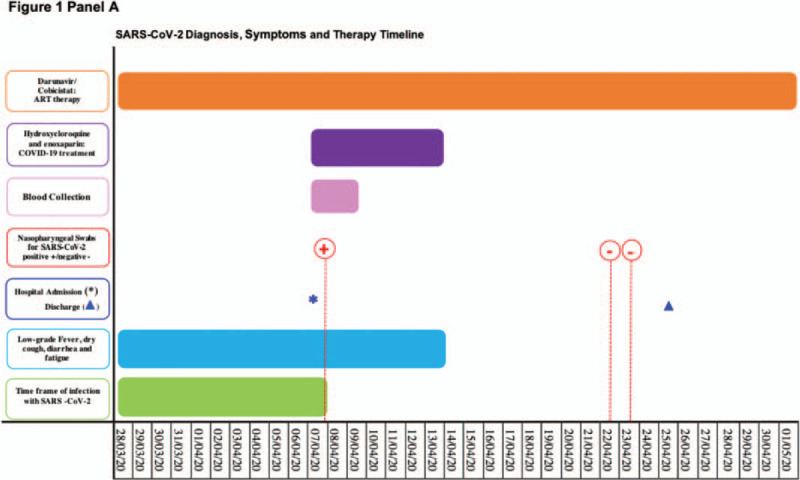


## References

[1] d’Ettore G Recchia G Ridolfi M Analysis of type I IFN response and T cell activation in severe COVID-19/HIV-1 coinfection: A case report. *Medicine*. 2020 99:e21803. 32899009 10.1097/MD.0000000000021803 PMC7478511

